# Psychiatric Manifestations in a Young Man with Osteogenesis Imperfecta and Family History of Schizophrenia: A Case Report

**DOI:** 10.1192/j.eurpsy.2026.12144

**Published:** 2026-07-31

**Authors:** M. Oveysi Kian, E. Shahsavand Ananloo

**Affiliations:** 1 Department of Psychiatry, University of Rehabilitation and Social Health Sciences; 2 Department Psychiatry School of Medicine, Tehran University of Medical Sciences (TUMS), Tehran, Iran, Islamic Republic Of

## Abstract

**Introduction:**

Osteogenesis Imperfecta (OI) is a congenital connective tissue disorder caused by abnormalities in type I collagen, leading to bone fragility and recurrent fractures.

This report describes a young man with OI and a strong family history of schizophrenia who exhibited a developmental progression of psychiatric symptoms—from childhood hyperactivity and obsessive traits to the onset of psychosis in early adulthood. This case highlights the need for early psychiatric monitoring and coordinated multidisciplinary care in patients with OI who present with behavioral or emotional changes.

**Objectives:**

The objective of this report is to demonstrate how biological vulnerability, developmental factors, and psychosocial stressors can interact to produce psychosis in a patient with Osteogenesis Imperfecta (OI). We describe the progression of symptoms from childhood behavioral difficulties to psychosis in early adulthood, examine the influence of family history and chronic illness, and emphasize the need for ongoing psychiatric monitoring and integrated multidisciplinary care in medically complex patients.

**Methods:**

This case was analyzed through retrospective review of hospital records, outpatient follow-up documentation, and radiologic findings. Data collection included demographic details, developmental and family history, psychiatric evaluations, and pharmacological interventions. MRI brain without contrast was performed to rule out structural or ischemic abnormalities.

The patient’s symptom trajectory was mapped across developmental stages to evaluate patterns of progression and remission. All psychiatric diagnoses were made according to DSM-5 criteria, and treatment response was assessed clinically over several months.

**Results:**

The patient is a 25-year-old male with a strong hereditary vulnerability, as his father has schizophrenia and his mother has obsessive-compulsive disorder. He was diagnosed with Osteogenesis Imperfecta at 35 days of age following a complete bone fracture and received early treatment with Alendronate, Calcium, and Vitamin D, though no ongoing therapy continued into adulthood.

This case shows how genetic risk, chronic physical illness, and developmental stress can interact to produce psychosis in OI.

**Image 1:**
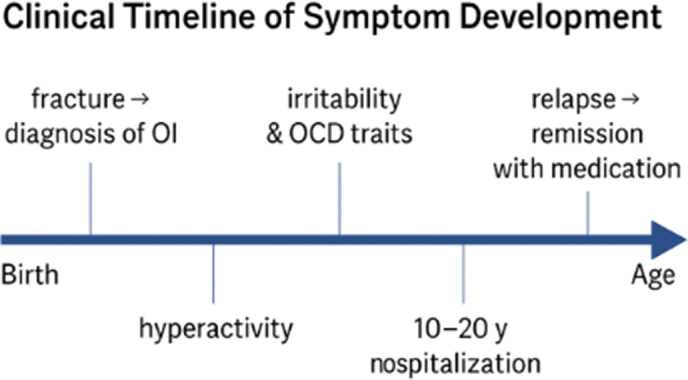

**Conclusions:**

This case demonstrates that psychiatric manifestations in Osteogenesis Imperfecta (OI) can arise from a multifactorial interaction between genetic predisposition, neurodevelopmental challenges, and chronic psychosocial stress. Even in the absence of neuroimaging abnormalities, patients with OI may show vulnerability to severe psychiatric disorders such as psychosis. Early psychiatric assessment, consistent pharmacological treatment, and long-term multidisciplinary follow-up are essential for stabilizing mental health and improving quality of life in patients with chronic congenital conditions like OI.

**Disclosure of Interest:**

None Declared

